# MRgLITT under general anesthesia using extended non-ferromagnetic components in a non-MR-compatible environment-a case report

**DOI:** 10.1186/s12871-026-03692-4

**Published:** 2026-02-12

**Authors:** Zhijing Yang, Ruibo Zhang, Yi Su, Lu Li, Long Chen, Yong Chen, Jinqi Lin, Yan Huang, Jun Xiong

**Affiliations:** 1 Department of Anesthesiology, Shenzhen University General Hospital, Shenzhen University, No. 1098 Xueyuan AVE., Xili, Nanshan District, Shenzhen, Guangdong Province 518055 China; 2 Functional Neurology and Neurosurgical Center, Shenzhen University General Hospital, Shenzhen University, No. 1098 Xueyuan AVE., Xili, Nanshan District, Shenzhen, Guangdong Province 518055 China; 3 School of Biomedical Engineering, Shenzhen University Medical School, Shenzhen University, Xueyuan Avenue, Nanshan District, Shenzhen, Guangdong Province 518055 China; 4 Operation Center, Shenzhen University General Hospital, Shenzhen University, No. 1098 Xueyuan AVE., Xili, Nanshan District, Shenzhen, Guangdong Province 518055 China

**Keywords:** General anesthesia, Magnetic resonance imaging, Laser interstitial thermal therapy, Non-ferromagnetic circuit, Patient safety

## Abstract

**Background:**

Magnetic resonance-guided laser interstitial thermal therapy (MRgLITT) is a novel minimally invasive treatment that requires precise implementation within a magnetic resonance (MR) environment. Given the procedure’s lengthy duration and the need for absolute immobility, general anesthesia (GA) with endotracheal intubation (ETT) and an MR-compatible ventilator is recommended. However, in facilities lacking a hybrid MR operating room or MR-compatible anesthesia equipment, alternative anesthesia strategies must be employed to ensure patient safety while complying with American Society of Anesthesiologists (ASA) recommendations for sedation and GA in the MRI environment, including monitoring of heart rate, pulse oximetry, and blood pressure.

**Case presentation:**

A 43-year-old male ASA II patient, with epilepsy and cavernous hemangioma, was scheduled for elective MRgLITT. The critical second phase of this procedure required GA with ETT within the MRI environment. Due to the absence of MR-conditional anesthesia equipment in the MR suite, extended-length, MR-safe components were adapted from existing equipment. These modifications included a prolonged breathing circuit, an extended-length cable for the pulse oximeter probe, an extended air-conduction tube for non-invasive blood pressure (NIBP), and a prolonged sampling line for the capnograph. The effectiveness and safety of these adapted devices were verified against standard monitoring equipment and a conventional anesthesia machine in the pre-procedural test conducted during the first phase of MRgLITT. Furthermore, arterial blood gas analysis provided additional confirmation of patient safety using the modified hardware. By using these extended lines, conventional ventilators and monitors can be positioned outside the MR scanner room, enabling continuous support and monitoring of the patient’s vital signs throughout the procedure. The patient was very stable intraoperatively, particularly in the MR suite, which required extended equipment. Ultimately, the MRgLITT was successfully completed using this innovative anesthesia management approach. The patient recovered very quickly.

**Conclusion:**

In the absence of MR-compatible equipment, adapted non-ferrous devices with extended-length components can provide necessary physiologic support and monitoring, aligning with ASA guidelines for sedation and GA in an MRI environment. However, rigorous pre-procedural testing of such modified equipment is essential to ensure patient safety. The innovative anesthesia approach we implemented for MR-guided procedures demonstrates clinical utility and offers a practical model for anesthesiologists managing similar resource-constrained scenarios.

## Background

The therapy of drug-resistant epilepsy has to depend on surgical methods. Some seizure types can be treated effectively by traditional surgical techniques or the less invasive laser ablation [1]. Magnetic resonance-guided laser interstitial thermal therapy (MRgLITT) is a novel minimally invasive technique used to treat various intracranial conditions such as drug-resistant epilepsy, brain metastases, gliomas, and radiation necrosis. This technique uses a laser probe to heat intracranial lesion tissue, achieving thermal ablation. The whole treatment procedure and temperature are controlled under continuous real-time magnetic resonance image (MRI) guidance, which may also avoid the surgical trauma associated with traditional craniotomy.

Patients undergoing MRgLITT first undergo intracranial placement of a fiber-optic probe, which is routinely performed in the operating room under anesthesia. They are then transferred to the MRI suite for subsequent scanning and magnetic resonance (MR) guided laser ablation. Throughout this lengthy procedure, absolute immobility is mandatory. Given the requirement for complete stillness and the need for secure airway management, sedation alone is insufficient. Consequently, general anesthesia (GA) with endotracheal intubation (ETT) is the optimal anesthetic management for MRgLITT.

GA with ETT in the MRI environment presents unique challenges not encountered in the operating room. Consequently, it is imperative to maintain the same level of safety and monitoring as in the operating room, as described in [2, 3]. To ensure patient safety, MRgLITT should therefore be conducted either in a hybrid MR operating room or using MR-compatible ventilators and monitors. For example, in Japan, the monitors in the MR suite should be MR-compatible [4]. These equipment and technique requirements increase the challenges of GA management in MR environments without MR-compatible devices.

As the importance of MRgLITT treatment and insufficient preparation time, establishing a hybrid MR operating room or purchasing MR conditional equipment was prohibitively expensive and unrealistic. Meanwhile, there were limited number of reports from non-MR-compatible centers, thus we had to adapt existing devices to create a suitable alternative ventilator and monitoring system for this treatment.

Now, the case was presented to describe the anesthetic management and technical adaptations that enabled safe MRgLITT under GA in a non-MR-compatible setting.

## Case presentation

A 43-year-old male patient, American Society of Anesthesiologists (ASA) II, was admitted with symptomatic epilepsy and cavernous hemangioma. Despite undergoing three gastrointestinal surgeries between July 2022 and March 2025, he maintained health apart from troublesome epilepsy symptoms that began in January 2023. His body mass index was 19.29, and his airway was normal; preoperative laboratory tests were normal. Based on clinical presentation, electroencephalogram, and head MRI results, his diagnosis was confirmed, and a treatment plan was finally established. Given the presence of a right amygdala cavernous hemangioma and drug-resistant symptoms presumed to be the epilepsy focus, he was scheduled for MRgLITT in two weeks. Because of significant minimal trauma and quick recovery, MRgLITT was considered to be the most appropriate.

### Preparation

**Fig. 1:**
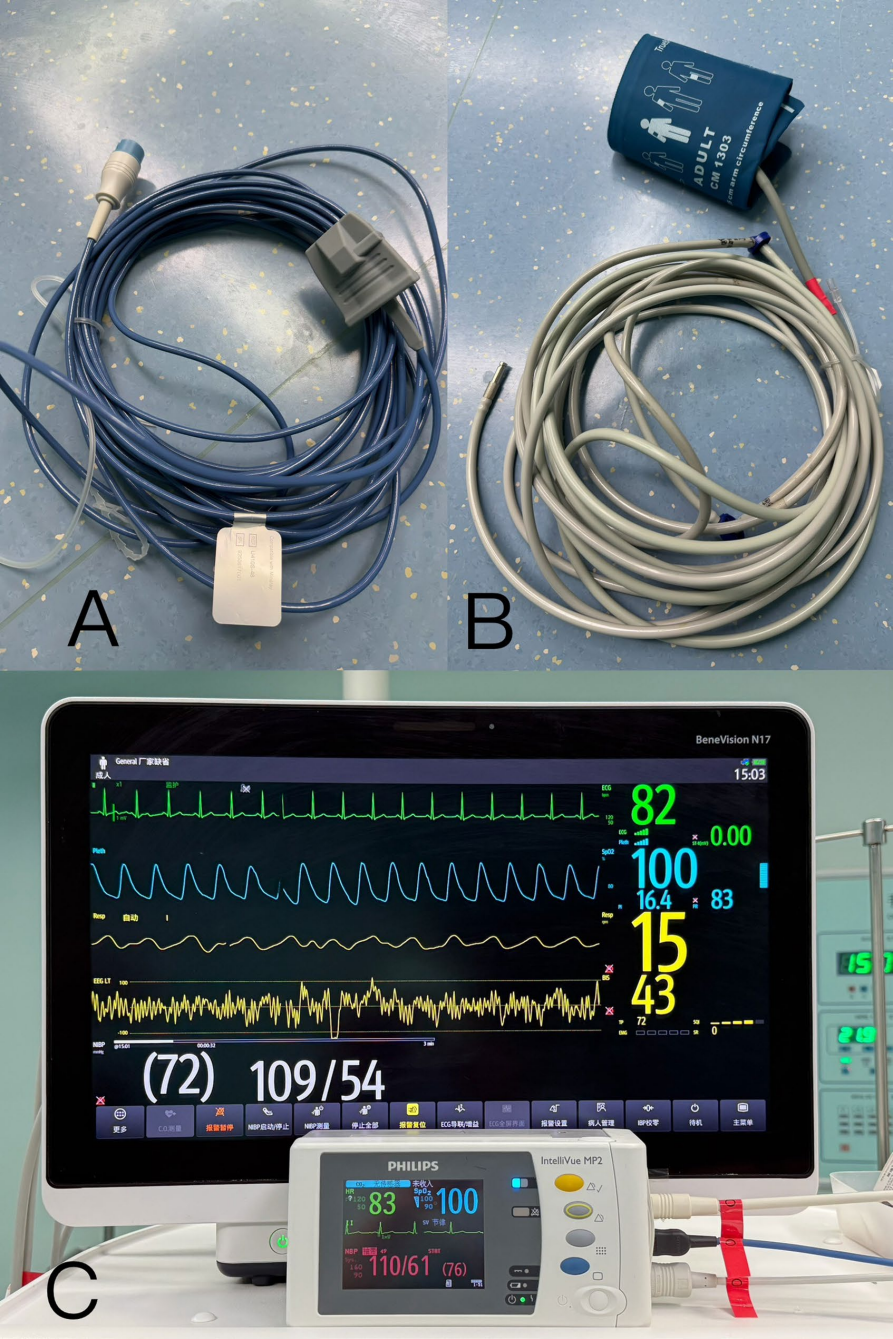
**A** the extended pulse oximeter. **B **the extended air-conduction tube for NIBP. **C **two independent sets of NIBP and SpO2 (Philips IntelliVue MX800 and Mindray BeneVision N17) were simultaneously recorded and compared

**Fig. 2:**
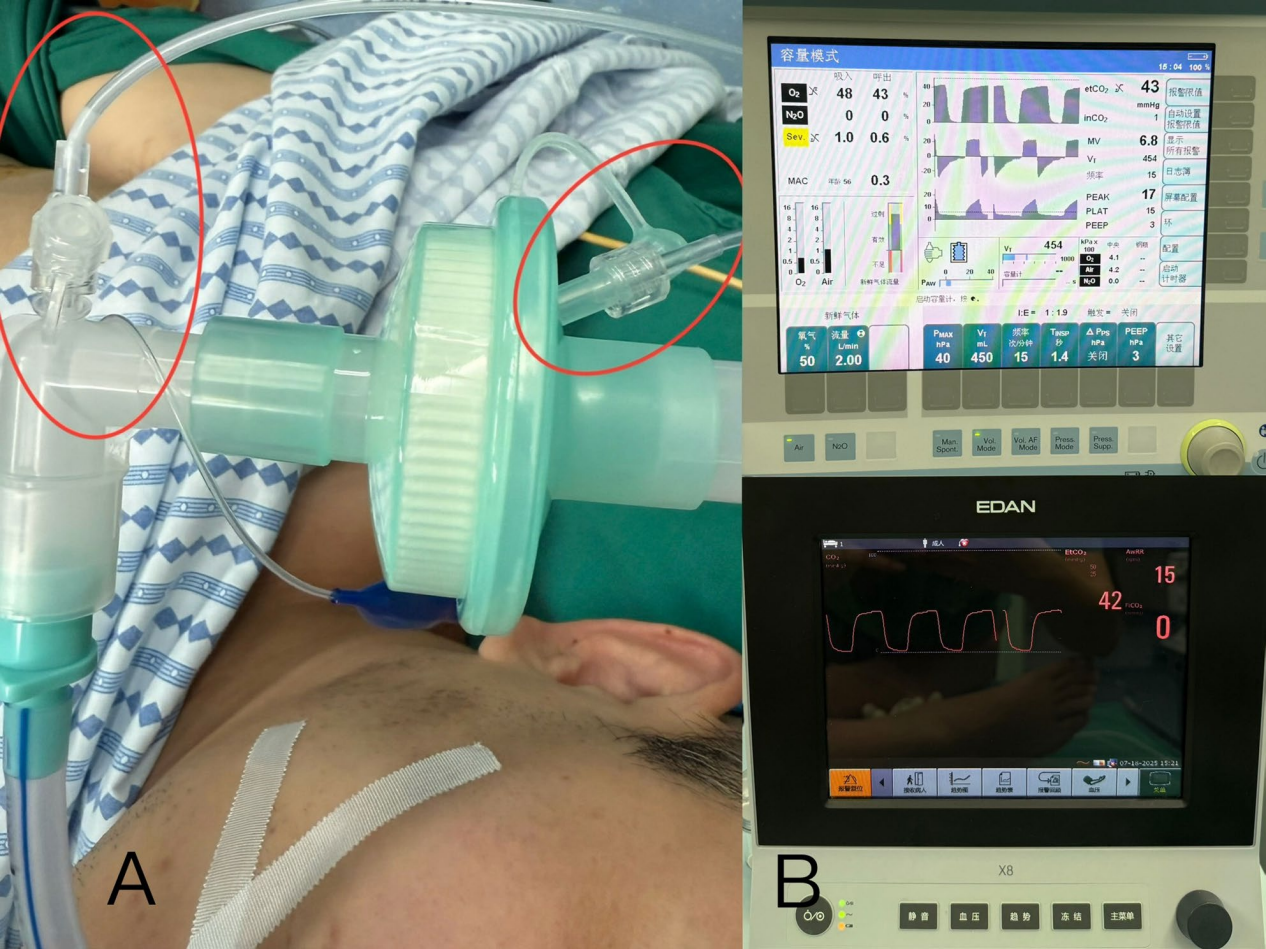
**A **the standard and extended sampling lines for exhaled carbon dioxide concentration (EtCO2) monitoring connected to the oral end of endotracheal tube. **B **two sets of EtCO2 monitoring. Drager Primus IE anesthesia workstation and EDAN X12 monitor showed EtCO2 43mmHg and 42mmHg respectively

Upon arrival in the operating theatre, standard ASA monitoring (Philips IntelliVue MX800, Philips, Suzhou, China) was applied to the patient’s right upper arm, including non-invasive blood pressure (NIBP), pulse oximetry (SpO_2_), electrocardiography (ECG), and body temperature. Simultaneously, another monitoring system (Mindray BeneVision N17, Shenzhen Mindray Biomedical Co., Ltd., Shenzhen, China) was connected via a nine-meter extension to his left upper arm for SpO_2_ and NIBP measurements. The extended NIBP air-conduct tube consisted of four standard-length segments connected in series (Fig. 1). For exhaled carbon dioxide concentration (EtCO2) monitoring, two sampling lines were connected to the oral end of the endotracheal tube, a standard-length line to the Drager Primus IE anesthesia workstation (Draeger Medical Systems (Shanghai) Co., Ltd. Shanghai, China), another nine-meter extended one to EDAN X12 monitor (EDAN Medical, Shenzhen, China) (Fig. 2).

### Anesthesia induction and maintenance

After intravenous access with a 20-G cannula was secured, GA induction was performed after adequate pre-oxygenation. Sulfentanil was administered 20 µg, ciprofol 25 mg, dexamethasone 5 mg, and rocuronium 50 mg. When loss of consciousness occurred, assisted ventilation was applied. ETT was established with a non-ferrous tube after confirming muscle relaxation.

GA was maintained with remifentanil 0.1 ~ 0.15 µg/kg/min, propofol 3 ~ 7 mg/kg/h, and sevoflurane 0.5%~1.5% based on bispectral index (45 ~ 65) and the change trends of HR and NIBP. The ventilation setting included the tidal volume of 450 ml, the ratio of inspiration and expiration of 1:2, the respiratory rate of 15 breaths per minute to keep the EtCO2 range of 35 ~ 45 mmHg, and 50% oxygen/air mixture, and the fresh gas flow of 2 L/min.

### Testing the safety and availability of this modified equipment

**Fig. 3:**
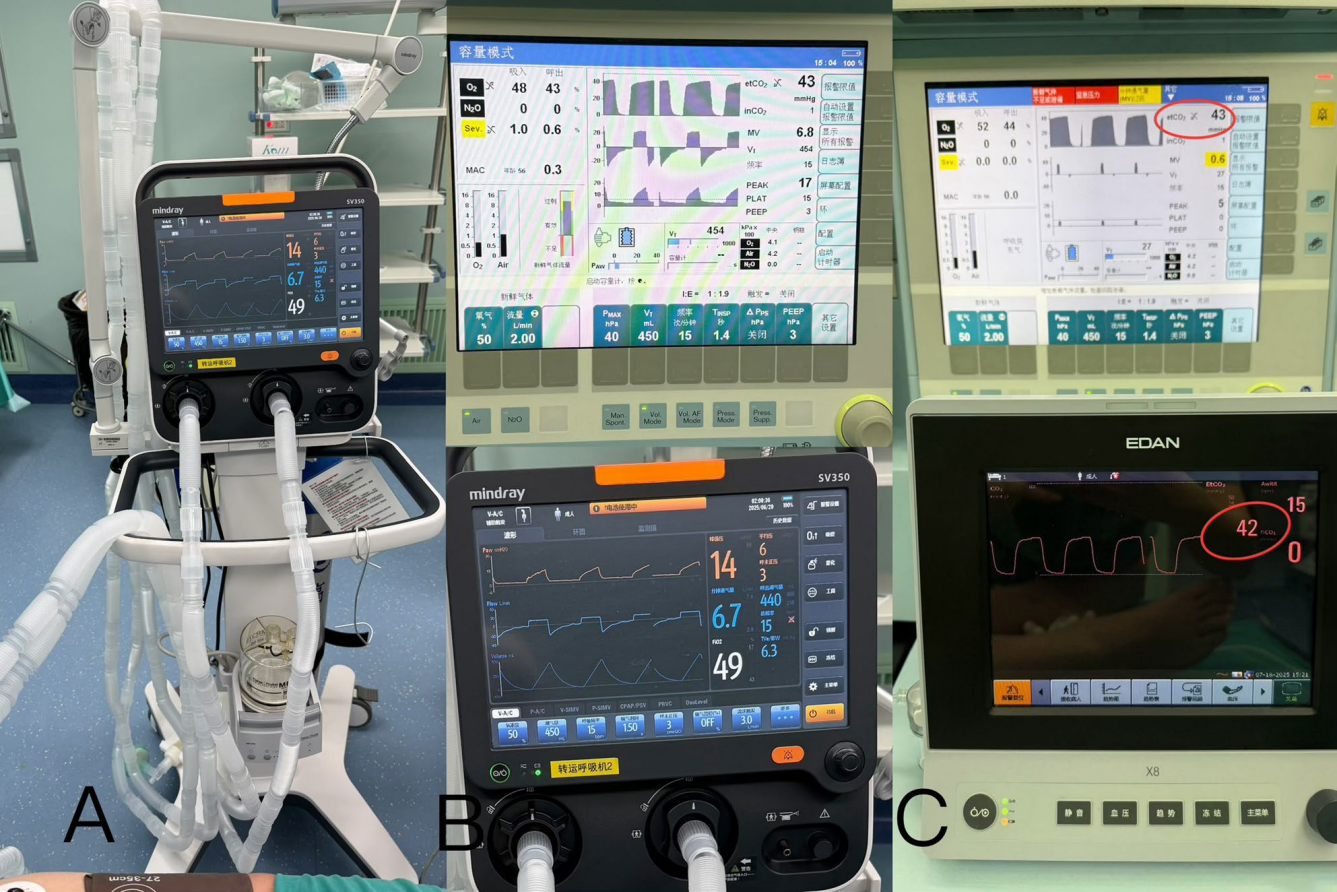
**A **the respiration support was switch to the transport ventilator connected with the extended breath circuit. **B **the ventilation settings on the anesthesia workstation and the transport ventilator. **C **the comparison of EtCO2 under the transport ventilator support

Following positioning, two laser fiberoptics were inserted intracranially using stereotactic guidance. This step was performed under GA routinely. Throughout the procedure, two independent sets of NIBP, SpO_2,_ and EtCO2 were simultaneously recorded and compared. Arterial blood gas analysis was also performed to evaluate the adequacy of ventilation. Concurrently, a backup transport ventilator (Mindray SV350, Shenzhen Mindray Biomedical Co., Ltd., Shenzhen, China) with a nine-meter anesthesia circuit was prepared and initiated following a successful self-test (Fig. 3). Upon completion of laser probe insertion, inhalation anesthesia was discontinued. GA was subsequently maintained exclusively via total intravenous anesthesia. And the ventilator circuit was switched to the transport ventilator. The patient’s vital signs were continuously monitored using both sets of equipment until departure from the operating room.

### Transfer to the MRI suite

**Fig. 4:**
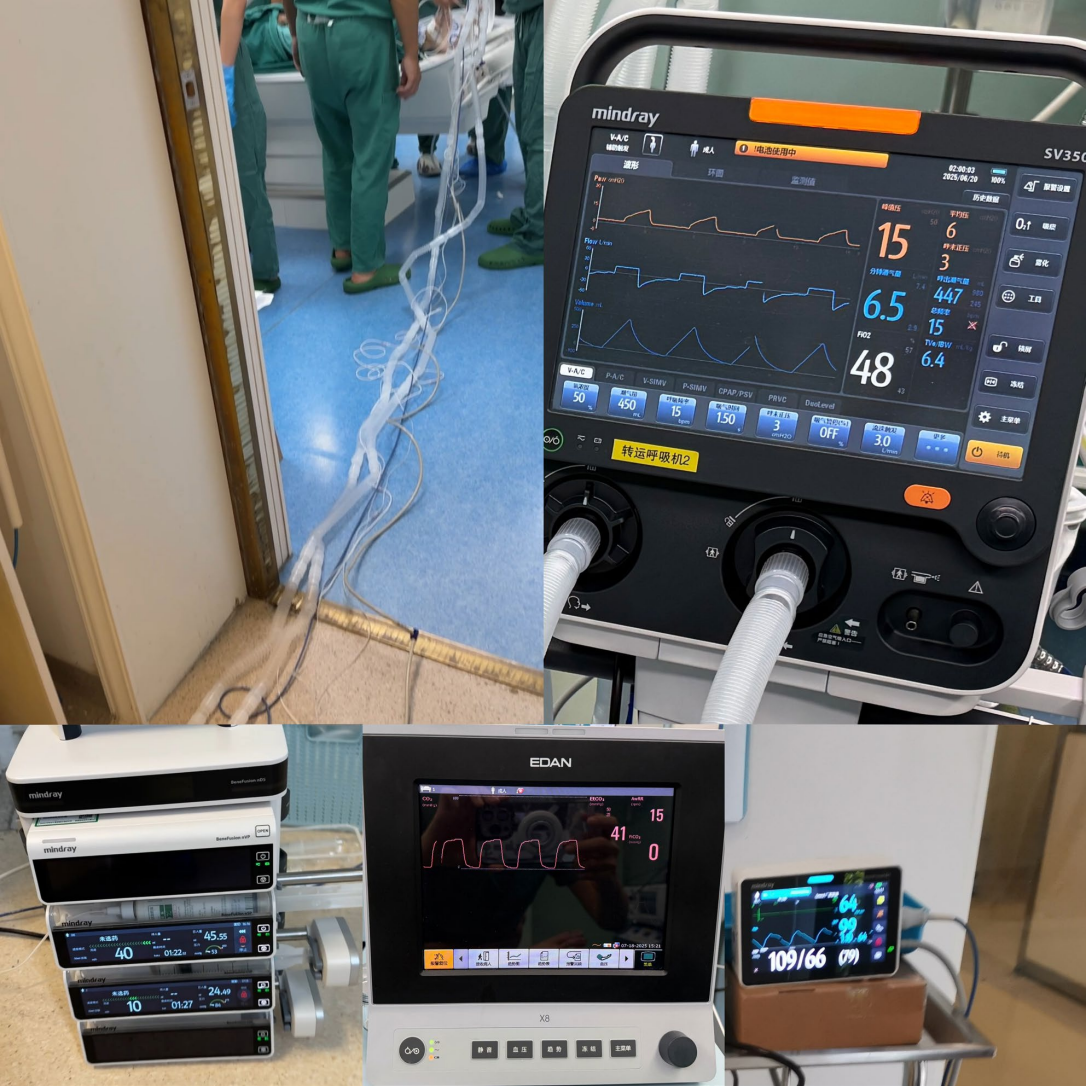
The transport ventilator (Mindray SV350), infusion pumps, and the monitoring of EtCO2 (EDAN X12), SpO2 and NIBP (Philips IntelliVue MX800) were placed outsidethe MRI suite. The upper part of the figure, the extended tubing and circuits connected with patient in a linear manner

Two and a half hours after GA, following careful preparation, the patient was transported to the MRI suite. Upon leaving the operating room, NIBP, SpO2, and EtCO2 were monitored solely using the elongated Mindray BeneVision N17 and EDAN X12 monitor systems. Ventilatory support was provided by the Mindray SV350 transport ventilator connected via a nine-meter anesthesia circuit. Ventilator settings remained identical to those used in the operating room. To maintain intravenous anesthesia, including propofol and remifentanil, eight extension infusion tubes were connected in series, forming a prolonged infusion line linking the patient to the infusion pump system. These specially engineered extended tubing and circuit enabled the non-MR-compatible equipment to be safely operated outside the MRI scanner enclosure, far beyond the 5-gauss line (Fig. 4). Although these tubes and circuits are made of non-metallic materials, they were arranged linearly to avoid forming loops and overheating. The second arterial blood gas analysis confirmed equivalent ventilation efficacy between the standard and extended-length anesthesia circuits (Table 1).

**Table 1:** Twice arterial blood gas analyses of the standard circuit and anesthesia machine, and the extended circuit and transportation ventilator

	Standard circuit/anesthesia machine	Extended circuit/transport ventilator		Standard circuit/anesthesia machine	Extended circuit/transport ventilator
PH	7.38	7.40	Glu (mmol/L)	4.70	6.4
PCO_2_ (mmHg)	45	44	Lac (mmol/L)	0.8	0.9
PO_2_ (mmHg)	232	254	Hb (g/L)	118	108
Hct (%)	33	37	SO_2_ (%)	98.5	99.0
Na^+^ (mmol/L)	136	136	HCO_3_^−^ (mmol/L)	26.6	27.3
K^+^ (mmol/L)	4.10	4.7	BE	1.5	2.5
Ca^2+^ (mmol/L)	1.21	1.25	Cl^−^ (mmol/L)	105	106

### Recovery

**Fig. 5:**
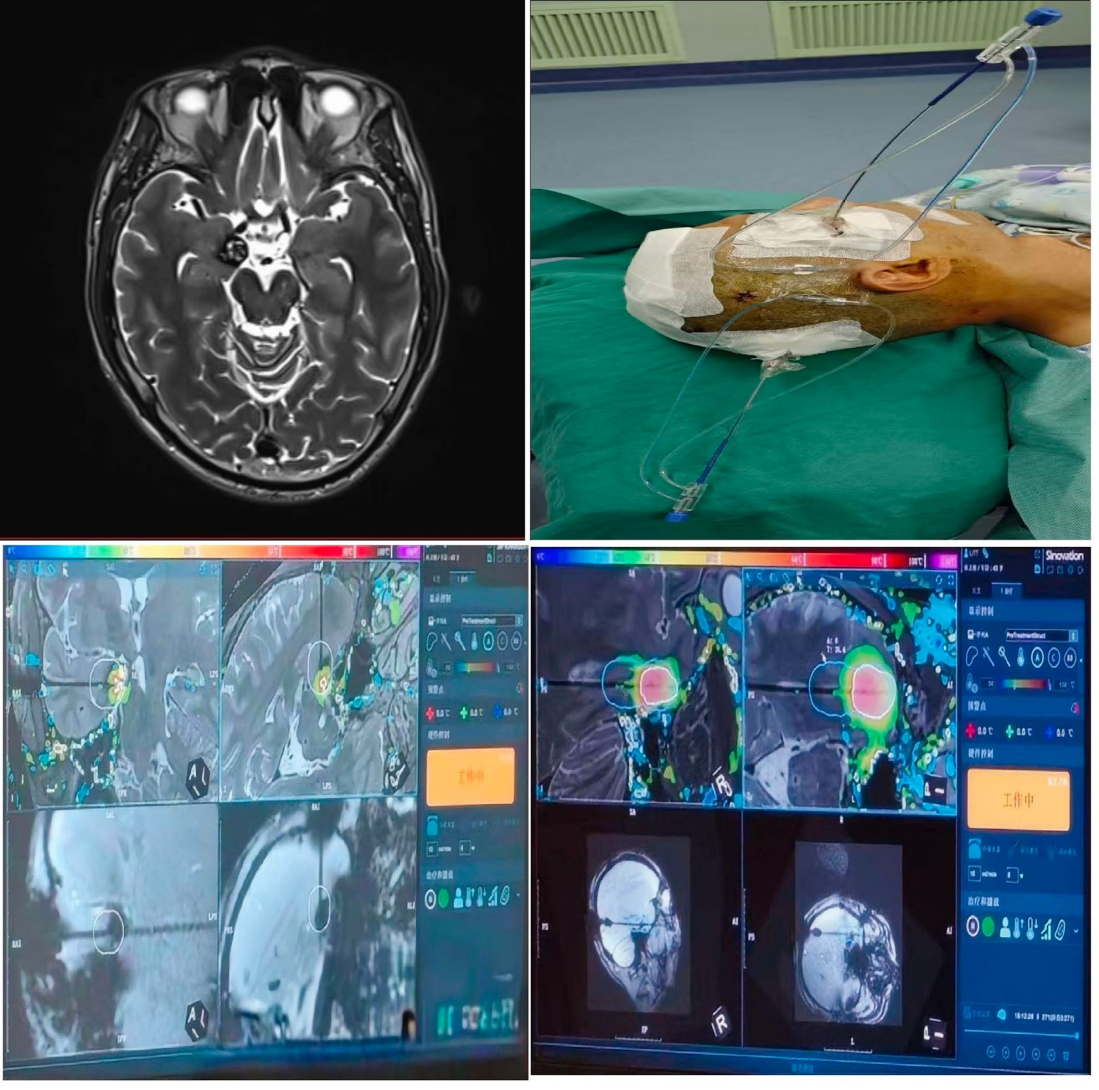
The MR image of intracranial lesion, the patient with two intracranial fiber-optic probes, and the images of MR guided treatment

Three hours later, real-time MRI demonstrated successful MRgLITT. The patient was transported back to the operating room and reconnected to the standard monitor system and anesthesia workstation. The two intracranial laser probes were then removed, and the wound was closed (Fig. 5). After reversal of neuromuscular blockade and thorough suction of the airway and oropharynx, the patient was extubated, achieving full return of consciousness and demonstrating adequate protective reflexes. Throughout the procedure, from the operating room to the MR suite and returning to the operating room, the patient maintained stable vital signs, and postoperative recovery was uneventful.

## Discussion

For this patient, GA with ETT was considered relatively safe and effective, primarily because complete immobility was required. Any head movement could not only damage the precision laser probe but also degrade MRI image quality, thereby compromising therapeutic efficacy. Furthermore, inherent characteristics of MRI, such as significant acoustic noise and prolonged scan time, can limit patients’ ability to tolerate and complete the examination [5].

Although our team is experienced in providing sedation within the MRI environment, significant concerns persisted regarding the risks of respiratory support and monitoring in this case. This was due to the absence of an MR-compatible operating room and of MR-conditional monitoring devices. Furthermore, acquiring MR-compatible monitors and an anesthesia machine was not feasible temporarily. Nevertheless, to ensure the safe and effective administration of anesthesia in the MRI unit, ASA standard intraoperative monitoring items are recommended [6, 7]. In light of constraints, we leveraged existing devices to develop extended configurations for ventilation and monitoring, thereby reducing the risk of adverse events.

The fiberoptic pulse oximeter was essential for detecting hypoxia and providing pulse rate data, which offered partial cardiac rhythm information in lieu of ECG monitoring [8]. Given the absence of MR-conditional ECG electrodes, conventional ECG monitoring was necessarily abandoned instead by a pulse oximeter, whose safety within the MRI suite was confirmed preoperatively. Although it is physically incapable of causing a burn [8], we placed an ice pack over the probe site during MRgLITT as an additional precaution.

NIBP monitoring and capnography were readily established using plastic air-conduction tubes and sampling lines, thereby avoiding ferromagnetic components. The more significant challenge involved the length of these conduits. An extended NIBP air-conduction tube with higher compliance can introduce measurement error [9]. Similarly, excessive length or volume in the capnography sampling line also affects gas transit, potentially compromising waveform accuracy and EtCO_2_ readings [10]. Consequently, we minimized the lengths of both the NIBP and capnography circuits to the greatest extent feasible, given the distance between the MRI scanner and the controlled-access area, to mitigate these errors. This necessity for optimized accuracy directly explained the use of dual pulse oximeter, NIBP, and EtCO_2_ monitoring systems in the operating theatre.

The extended anesthesia circuit supporting ventilation also faced challenges related to increased compliance. Alterations in breathing-circuit length and compliance can lead to inaccurate delivery of the desired tidal volume [11]. To address this, we minimized the circuit length. Prior to clinical use, the functionality of the nine-meter circuit was verified by testing the transport ventilator with a lung simulator. All ventilation parameters were matched to those of the primary Drager Primus IE anesthesia workstation. At the end of the first stage of MRgLITT, ventilation was successfully switched to the transport ventilator. No significant difference in EtCO_2_ was observed between ventilator systems. Twice arterial blood gas analyses confirmed comparable ventilation efficacy between standard and extended-length circuits.

### Limitations

During the second MRgLITT phase, ECG monitoring was discontinued due to the unavailability of non-ferromagnetic leads. Concurrently, core body temperature typically decreases under GA, particularly in a cold scanning room. However, continuous temperature monitoring was precluded by an insufficiently long probe cable. Although prolonged MRI examination may elevate body temperature [12], the patient received active warming and immediate temperature monitoring upon returning to the operating room for anesthetic recovery, given the MRI suite’s typically lower ambient temperature.

In summary, administering GA with ETT in the MRI suite is significantly challenging, particularly without specialized MR-compatible equipment, thereby complicating the procedure. We successfully performed the inaugural MRgLITT under GA by implementing adapted ventilator methods and strategically deploying conditional monitors to maintain safety standards (Table 2). Our experience shows that modifying existing equipment and carefully selected monitoring can enable safe MRgLITT execution. Additionally, some practices had to be designed by our personal experiences and modified according to the tricky situation, for example, using a pulse oximeter instead of an ECG and placing an ice pack. Finally, a checklist should be recommended for patients’ safety before and after their transfer and testing equipment, in accordance with the patient-centered concept.

**Table 2:** Key challenges of ventilation and monitoring of no-MR-compatible equipment in the MR environment

	Challenges	Clinical Solution
Extended ventilation circuit	Dead space increment	Regulating ventilation setting based on the standard anesthesia machine, monitoring EtCO_2_ and arterial blood gas analysis
Extended EtCO_2_ sampling line	Delay or damping of signals or waveform	Pretest the extended sampling line and compare with the standard line
Extended NIBP	Accuracy reduction	Compare with the standard monitor
ECG	No MR-compatible electrode	Instead with pulse oximetry partly
Body temperature	No MR-compatible probe	No body temperature monitoring in this case
Infusion line	Not long enough	Connecting eight infusion lines

## Conclusion

In MRI-guided procedures where compatible anesthesia equipment is unavailable, extended non-ferromagnetic circuits and monitoring components can ensure safe and effective anesthesia if rigorously tested beforehand. This approach offers a practical model for anesthesiologists in similar conditions. Further multi-case experiences are needed to standardize anesthesia management for MR-guided procedures in resource-limited environments.

## Data Availability

No datasets were generated or analysed during the current study.

## Abbreviations

ASA: American Society of Anesthesiologists
ECG: Electrocardiography
ETT: Endotracheal intubation
EtCO2: Exhaled carbon dioxide concentration
GA: General anesthesia
MR: Magnetic resonance
MRgLITT: Magnetic resonance-guided laser interstitial thermal therapy
MRI: Magnetic resonance imaging
NIBP: Non-invasive blood pressure
